# Looking Back on Digital Medical Education Over the Last 25 Years and Looking to the Future: Narrative Review

**DOI:** 10.2196/60312

**Published:** 2024-12-19

**Authors:** Oluwadamilola Ogundiya, Thahmina Jasmine Rahman, Ioan Valnarov-Boulter, Tim Michael Young

**Affiliations:** 1 Queen Square Institute of Neurology University College London London United Kingdom

**Keywords:** digital health, digital medical education, health education, medical education, mobile phone, artificial intelligence, AI

## Abstract

**Background:**

The last 25 years have seen enormous progression in digital technologies across the whole of the health service, including health education. The rapid evolution and use of web-based and digital techniques have been significantly transforming this field since the beginning of the new millennium. These advancements continue to progress swiftly, even more so after the COVID-19 pandemic.

**Objective:**

This narrative review aims to outline and discuss the developments that have taken place in digital medical education across the defined time frame. In addition, evidence for potential opportunities and challenges facing digital medical education in the near future was collated for analysis.

**Methods:**

Literature reviews were conducted using PubMed, Web of Science Core Collection, Scopus, Google Scholar, and Embase. The participants and learners in this study included medical students, physicians in training or continuing professional development, nurses, paramedics, and patients.

**Results:**

Evidence of the significant steps in the development of digital medical education in the past 25 years was presented and analyzed in terms of application, impact, and implications for the future. The results were grouped into the following themes for discussion: learning management systems; telemedicine (in digital medical education); mobile health; big data analytics; the metaverse, augmented reality, and virtual reality; the COVID-19 pandemic; artificial intelligence; and ethics and cybersecurity.

**Conclusions:**

Major changes and developments in digital medical education have occurred from around the start of the new millennium. Key steps in this journey include technical developments in teleconferencing and learning management systems, along with a marked increase in mobile device use for accessing learning over this time. While the pace of evolution in digital medical education accelerated during the COVID-19 pandemic, further rapid progress has continued since the resolution of the pandemic. Many of these changes are currently being widely used in health education and other fields, such as augmented reality, virtual reality, and artificial intelligence, providing significant future potential. The opportunities these technologies offer must be balanced against the associated challenges in areas such as cybersecurity, the integrity of web-based assessments, ethics, and issues of digital privacy to ensure that digital medical education continues to thrive in the future.

## Introduction

### Background

The last 25 years have seen striking digital technological advances in the internet, now accessed by many of us daily in everyday life through multiple devices, including smartphones. This period also provides an interesting unit of time over which changes in digital medical education can be viewed, as it spans a very significant portion of an individual health worker’s career. A medical student who started their studies in 1999 may now be an experienced consultant or general practitioner (GP). What purpose does looking back at such a time frame have for us now? While research is typically forward looking, knowledge acquisition is not an inexorable upward curve of progress. Knowledge can be lost unless we look for it. Without deliberate reflection, valuable insights can be lost in the rapid pace of discovery.

Historically, knowledge acquisition has not always followed an inexorable upward curve of progress. In 1901, a strange device, the Antikythera mechanism, was retrieved from an ancient sunken Roman shipwreck just off the Greek island of Antikythera [1]. This complicated, geared analog computer was capable of predicting planetary movements and eclipses. It was >2000 years old with no known precursor, and nothing near its complexity would be produced for >1000 years. The invention and its secrets were lost to history in the shipwreck. In our modern era, despite vast stores of information, we may still face similar risks. For example, entering the search term *digital medical education* on Google Scholar, restricted to 2023 to 2024, yields >1300 articles. Thus, new discoveries run the risk of being submerged under a sea of data, justifying reviews that provide a contemporaneous overview of digital medical education and identify areas for further progress, such as this one. Therefore, it is necessary to examine the ever-changing state of digital medical education, including the emerging technologies that help define this era.

While digital health concerns the use of technology for improving health and well-being [2], the term *digital medical education*, as used in our study, transcends this definition to focus instead on the use of digital technologies in medical education. In contrast to traditional teaching methods, digital medical education leverages digital technologies, such as teleconferencing and learning management, to more efficiently create and manage web-based courses and allow the educational experience to span beyond the traditional four walls of the classroom.

**Figure 1:**
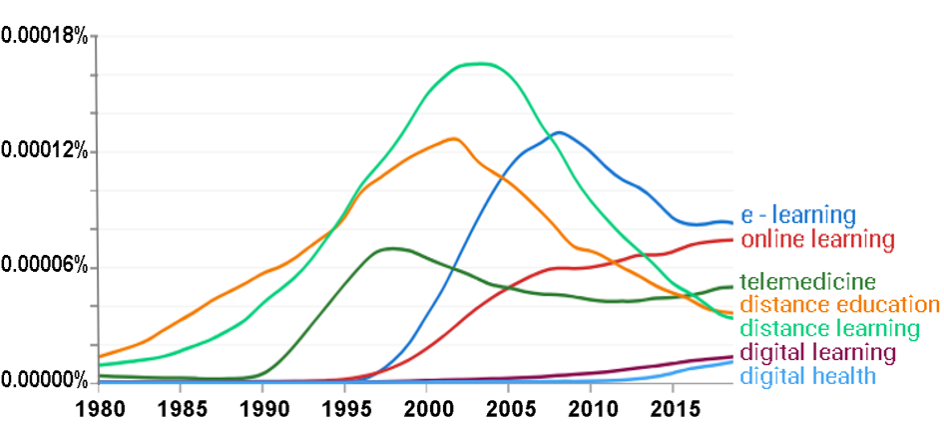
Frequency of the use of phrases related to e-learning by year. Google Ngram illustration showing the frequency of use by year as a percentage of appearance in the extensive corpus of scanned Google Books [5].

To contextualize the evolution of digital medical education, it is instructive to briefly consider the terminology underpinning it. Terms such as *distance learning*, *web-based learning*, *e-learning*, and *digital learning* have often been used interchangeably, but there are important differences, especially when considering their origins and development. For example, e-learning, although it may be carried out remotely or in person with a teacher, does not always necessitate an internet connection, as demonstrated by offline computer-based learning, which was prevalent in the late 20th century. By contrast, web-based learning is inherently dependent on a working internet connection. Originating from the digital encoding of signals, digital learning, initially described computer-based technologies, though its context expanded with the widespread popularity of digital televisions around the year 2000. Similarly, while the etymology of telemedicine implies the provision of medical care remotely [3,4], the term is also extensively applied to medical education. Looking back at the last few decades, we can see that such terms continue to be in a state of flux regarding the popularity of use. For example, in Figure 1 [5], the inflection point of popularity for the term *telemedicine* in the late 1990s coincides with the first appearance of the term *e-learning*, suggesting the latter may have evolved in part as a simple contraction of the former.

The effectiveness of digital medical education broadly seems to have equivalence to traditional face-to-face approaches [6]. Allied with this, the enormous and expanding economic importance is apparent. The global e-learning market, estimated at US $6.6 billion by the International Data Corporation in 2002, is believed to have grown to >30 times that value since then [7,8].

Distance learning, where the educator and learner are significantly separated in space, has a long background history of use. By the late 19th century, major universities provided degrees via distance learning, and by the early 20th century, the development of radio and the emergence of television allowed for new opportunities for distance learning. Programmed Logic for Automated Teaching Operations (PLATO) was the first computer-mediated learning environment, and thus, the first medium used for digital learning [9]. PLATO was developed as a working model in 1960, with a distance learning (>25 miles) app demonstrated using phone connections by the next year [9]. Learners at the University of Illinois using PLATO were able to not only create but also edit digital images, helped in part by the use of touch-sensitive plasma screens in 1966 [9]. The students taught via PLATO, in its very first simulation (a health education case of acute myocardial infarction), outperformed those taught with conventional face-to-face methods, and by the mid-60s, the first fully web-based university course was developed [9]. By 1984, the Open University, established in the United Kingdom in the late 1960s, provided education to 60,000 undergraduate students from their homes [10]. Through the use of the Cyclops computer system, students and tutors could interact via a teleconferencing network, displaying great success [10,11].

The evolution of digital learning accelerated with the introduction of the US Advanced Research Projects Agency Network (ARPANET) in 1969, which was a packet-switched computer network that linked different computers together [12]. Although the absolute precedence of ARPANET has been disputed, the development of ARPANET laid the foundations for what would become the internet [13]. Initially, ARPANET developed into facilitating connections between networks (“internets”) and then to *the* internet in the early 1980s. Subsequent global reach was achieved through the Web from the early 1990s onward, with web servers and web pages rapidly increasing in number. By the start of our designated review period, the Web (retrospectively termed Web 1.0) was characterized by its fixed search and read-only capabilities [14]. This initial static phase laid the groundwork for future innovations in digital education.

### Digital Medical Health in 1999

Before we cover the progress in digital medical education over the last 25 years, it would be helpful to first establish the baseline in 1999. Formal continuing medical education (CME) has been an important cornerstone in physician education for >50 years and can provide a useful surrogate marker when considering how widespread and extensive digital medical education is. By 1999, approximately 277,000 physicians in the United States (about a third of the total) were using the web, and 19% of CME credits were earned digitally, including CD-ROMS and web access [15]. While this suggests that digital delivery of CME was already quite common in 1999, this was largely delivered in a passive form using simple text or graphics. Marked global disparities of access were apparent; another article published that year found that the overwhelming majority of medical school websites mentioning CME were based in the United States, and only a quarter of identified CME websites offered web-based CMEs at that time [16]. Technical barriers, such as software and server problems, were common, with approximately 5% of the identified sites found to be inaccessible due to this [16]. Even for the accessible web-based CME sites, only a few gave immediate feedback to the user [16].

In an article in the first issue of JMIR, we found that the UK National Database of Telemedicine was launched just 2 months before 1999, providing information on a web page to anyone working in the field of telemedicine, including in education, even outside of the United Kingdom [17]. A study published in 1999 assessed the use of this web page and reported that there were only 120 visits a week to this national web page, despite it having been publicized extensively [17]. Rather than the real-time web-based learner feedback that we are used to today, the authors described an analysis of keywords used in search engines in an attempt to make their website easier for users to find [17].

Patient digital medical education also existed in 1999. For example, GPs in Scotland described their own customized patient-oriented websites [18]. The resultant internet documents were then published on servers located at their local health board, allowing viewing via National Health Service Scotland intranet servers. The educational material could also be viewed by patients via public servers if they had an internet connection. For these Scottish GPs, a template had to be modified specifically for their GP practice website, and a facilitator was required to be available on-site to help with any difficulties [18]. Elsewhere, limitations of digital medical education in 1999 were noted, including concerns about the great variability of graphics, response time, and the lack of visualization between students and educators [19,20].

Such articles were largely published as inspiring exceptions rather than reflecting the norm at that time. Matheson [15] noted in 1999 that “Only a small fraction of the revolutionary changes in medical education made possible by the World Wide Web have yet been realized.” Medical educators of that time considered the potential opportunities of the digital environment to rethink course structure and duration [21]. However, concerns about the unstoppable pace of change were also expressed as a “tsunami of change about to break over our heads” [21]. Such hopes and fears from the past can speak to us now as we look ahead to the rapid expansion of technologies, such as artificial intelligence (AI) and the metaverse, and in turn, contemplate the future of digital medical education.

Our study broadly encompassed digital health including web-based learning and e-learning and the concept of distance learning. In addition, studies involving blended (also known as hybrid) delivery, where health education is given as a mix of digital (typically distance) learning and traditional face-to-face teaching, were included. The scope included publications covering a broad range of health learners.

These groups were selected because they represent key stages in the continuum of medical education and reflect cohorts where the impact of digital medical education has been particularly significant. Furthermore, they include the professionals who play a pivotal role in the construction and delivery of health courses themselves and individuals who, as a result of rapid advancements in health care technologies, have become increasingly reliant on digital tools for education and the delivery of health care to patients. The review did not specifically focus on digital pedagogies that were beyond the scope of our study and that have been covered elsewhere [22].

### Aims

This study aimed to perform a literature search to identify relevant publications that covered digital medical education and its associated technologies over the last 25 years. The further aims were to analyze the results with a thematic approach as a narrative review, including hypotheses on future possible developments in digital medical education. This approach was used to provide an accessible overview of the evolving and wide-ranging field of digital medical education.

## Methods

In this narrative review, we considered technological and educational factors that have developed in digital medical education over the last 25 years using literature searches. The focus was on advancements in digital tools and teaching strategies that have influenced the effectiveness and delivery of digital medical education. Textbox 1 outlines the inclusion and exclusion criteria used for the literature search.

Literature reviews were conducted to support this review using PubMed, Web of Science Core Collection, Scopus, Google Scholar, and Embase. The first 3 databases offer a long search string length, while Embase has recently been shown to be associated with a high number of unique references in reviews. The biomedical and health science emphasis of PubMed and Embase is balanced by the broader scientific coverage of Web of Science, Scopus, and Google Scholar [23,24].

The original literature searches were conducted between April 16, 2024, and August 31, 2024. The review process was iterative, allowing the inclusion of additional sources as they emerged during the study search period with the specific search terms agreed by the authors. The collected literature was analyzed thematically to identify common themes and key developments, which are described and highlighted in this paper.

**Inclusion and exclusion criteria for literature searches used in this review.**

**Inclusion criteria**

- Participants from original articles
  Medical students
  Trainee physicians
  Physicians undergoing continuing professional development
  Nurses
  Paramedical staff
  Patients or members of the public
- Topics
  Digital medical education
  Health education
  Medical education
  e-Learning
  Telemedicine, distance learning, and web-based learning
- Publication dates of articles: from 1999 to 2024
- Language of publication: English
- Search terms
  “health education”
  “digital medical education”
  “medical education”
  “e-learning”
  “distance learning”
  “learning management systems”
  “artificial intelligence”
  “big data analytics”
  “augmented reality”
  “virtual reality”
  “metaverse”
  “mobile health”
  “COVID-19”
  “cybersecurity”
  “ethics” and “digital health”
  “ethics” or “digital health”
  “telemedicine”
  “online learning”

**Exclusion criteria**

- Topics: focus on digital pedagogy
- Publication dates of articles: before 1999
- Language of publication: non-English language

## Results

### Overview

The literature search yielded 128 articles, which were used in the study to develop the narrative review. The results aligned with at least 1 of 8 different themes. These were learning management systems (LMSs); telemedicine (in digital medical education); mobile health (mHealth); big data analytics (BDA); the metaverse, augmented reality (AR), and virtual reality (VR); the COVID-19 pandemic; AI; and ethics and cybersecurity. The results are presented and analyzed sequentially for each of these themed subgroups. For each theme, the results are used to hypothesize the potential relevance for the further development of digital medical education in the near future.

### LMSs and Digital Health Courses

LMSs, or web-based learning environments, consist of specialized e-learning software that facilitates comprehensive course management [25]. While the concept of LMS is not new, its use in higher education, including digital medical education, has markedly expanded over the last 25 years. LMS allows the automated coordination of digital educational courses and associated resources to deliver education in a web-based environment [25]. Practically, educational material can be produced, posted, and edited together with other resource materials, such as electronic journals [26]. In turn, the learners can submit work, which is then marked, the results collated, and feedback sent to the learners via the same platform. Learners increasingly rely on a broad spectrum of e-learning tools to supplement their education. LMSs provide centralized platforms that enable more efficient management of such educational content and digital resources, and by 2016, they were considered key components in digital medical education [25]. LMSs serve as a bridge between traditional teaching strategies and modern e-learning technologies, and their use has allowed entire courses to be structured and developed, providing the potential for large-scale open access to health education through massive open online courses (MOOCs) as well [27]. MOOCs were developed during the US Open Education Resources movement in the early 2000s [27]. They incorporate a decentralized open design, encouraging interaction between learners, and have seen rapid growth in the last 16 years [27]. Since 2008, the launch of MOOCs has built on the improvements seen in Web 2.0. MOOCs underwent rapid expansion from 2012 onward, with both Udacity and edX (education platforms led by an American organization, and Harvard and Massachusetts Institute of Technology, respectively, offering internet-based courses and degrees) founded that year [28].

The parallel evolution of the Web to Web 2.0 in 2004 has been of central importance to the further development of LMS, which allowed for read-and-write capabilities rather than read-only capabilities, making it much more interactive [14]. Initially, it was feared that this new, participatory version of the Web might threaten the dominance of LMS in digital education. Instead, this proved to be a key progression from a digital medical education perspective, improving dynamic communication among learners, their web-based courses, and their tutors. The gradual introduction of Web 3.0 (also termed Web3) over the last decade has been associated with a less centralized structure and allows greater use of more complex semantics by users without having to break these down into simpler components [14,28]. Web 3.0 also offers a more flexible schema of data storage and interactions between data [14]. While Web 1.0 was considered “read-only” and Web 2.0 was “read-write,” Web 3.0 has been described as “read-write-own,” allowing some degree of digital ownership [28].

The development of LMS from the early 2000s reflects academic concerns that web-based courses significantly increase educators’ workload and could only effectively handle ≤20 students per course [29]. However, LMSs have since evolved to support a larger number of students remotely, significantly increasing the capacity for distance learning while simultaneously reducing the overall workload for instructors. Examples of this include Canvas (created in 2002), Blackboard (2004), and Moodle (2011), the latter being an example of open-source software widely used in digital medical education [30]. Being open source, Moodle can run on most operating systems, including Macintosh, Windows, and Linux [26]. Likewise, Moodle’s flexibility allows educators to customize it to reflect the complexity and diversity of medical curricula, and alongside its ease of integration with external programs and cost-effectiveness, it is often the LMS of choice in medical education [31]. These platforms have revolutionized medical education by providing flexible, personalized, and interactive learning spaces that offer discussion forums for students, the setting of assessments, and a communication channel for students [31].

Videoconferencing using LMS allows for synchronous interactions across continents and real-time health care student feedback on questions, teaching, and even on the course itself [32]. The development of teleconferencing software, such as Skype (founded in 2003), Google Hangouts (2005), Zoom (2011), and Microsoft Teams (2017), has revolutionized the delivery of health education, offering increasingly sophisticated tools for remote, live, and 2-way interactions [29]. Beyond sharing the live classroom experience at a distance, teleconferencing has allowed for live streaming surgical procedures, grand rounds, and even the remote proctoring of medical assessments [32,33]. Teleconferencing software is primarily used for videoconferencing; however, other modalities include audioconferencing and audio-graphic teleconferencing**.** Disruptions to medical education practices during the COVID-19 pandemic highlighted the demand for videoconferencing tools, accelerating their adoption, which will be covered more comprehensively in the *Impact of the COVID-19 Pandemic on Digital Medical Education* subsection [34]. The use of videoconferencing and LMS digital platforms in digital medical education has evidence of effectiveness, with a systematic review published by the World Health Organization in 2015 finding that e-learning in medical education from the year 2000 was overall equivalent in terms of skill and knowledge acquisition compared to traditional face-to-face courses [35].

LMSs also offer health care students the ability to customize and adapt their educational experience by pursuing extra content for topics of interest or by engaging with content that better caters to their personal learning style [36]. These additionally provide flexibility for medical students by allowing the use of asynchronous learning materials, such as case studies, prerecorded lectures, and question banks, accommodating varied lifestyles and schedules [37]. These characteristics improve satisfaction and engagement with medical education when used alongside traditional teaching methods while also helping to build skills in autonomous regulation and independence when trying to meet learning goals [38]. Furthermore, it is easier to update and restructure content on LMS, keeping medical educational materials more current than traditional syllabi and textbooks, which cannot be readily reviewed regularly.

The interactive dimension of many health courses places a particular emphasis on enhancing interactions with students despite the web-based delivery. The evolution of LMS has played a significant role in the practice of blended learning, which has been adopted into the curriculum of many institutions for medical education [39]. Blended learning has been demonstrated to yield significantly better knowledge acquisition in health education compared to traditional techniques [40]. The use of e-learning may actually be more effective in the accrual of factual knowledge than the use of traditional approaches [41]. More clinical scenarios, such as examinations and communication skills, have traditionally been taught through direct patient interactions or simulation teaching, either with manikins or standardized patients (typically actors). However, more recently it has been shown that the use of LMS platforms can also effectively create web-based simulation training in health education [42].

Digital question banks on LMS and participatory e-learning content have become some of the most popular revision methods for medical students, with studies demonstrating that these may be used by up to 92% of students [43]. Many use the concept of gamification by using instantaneous feedback from quizzes and the idea of “streaks,” which encourage students to maintain daily engagement with the platform [44]. These features are thought to increase student motivation and make it easier to track and visualize progress. Furthermore, digital platforms may use competitive aspects, such as leaderboards, badges, points, and challenges, allowing medical students to compare their progress with each other and may make learning more enjoyable [45]. These e-learning materials have also demonstrated efficacy in the continued education of health professionals without any evidence that these methods of learning affect professional behaviors or patient outcomes [46]. However, a scoping review evaluating digital medical education for medical students between 2000 and 2019 found that such courses varied in terms of delivery and assessment, were mainly delivered as electives, and often lacked robust evaluation [47].

From a wider perspective, digital education platforms have demonstrated the potential to help reduce geographic and financial barriers to medical education by allowing educators to share knowledge across borders. There is evidence that LMS can improve educational opportunities for medical students in resource-constrained lower-income countries [48]; however, inequalities in internet connectivity, technology, and language barriers could potentially worsen the disparities in health knowledge. It is important that e-learning content aligns with medical curricula and is standardized. This is achieved by developing quality assurance and guidelines for these platforms through collaborations with accrediting institutional bodies [49].

Moreover, evidence suggests that medical students find e-learning platforms to be effective in improving understanding and are associated with high satisfaction scores [50]. Health care student satisfaction overall with LMS use in medical education appears to be high, with >94% of medical and nursing students recently showing satisfaction with the LMS used in China [50]. Therefore, it is likely that these digital platforms will continue to play an important role in the educational toolkit of modern-day health care students.

Looking forward, several areas for the development of existing LMSs used in medical health education can be identified and broadly classified into either technical or use opportunities. On the technical side, the interface of LMS currently used can be potentially confusing to both the learners and the educators themselves. More intuitive interface development of LMS interfaces could be used to counter this potential challenge [31]. Increasing the use of a single sign-in for both LMS and institutional emails would be in line with student feedback and could help with communication via the LMS themselves [25]. In a similar manner, installation and support systems to help users optimize their use of Moodle as a free open-source platform have been identified as areas that might practically be improved to help educators [31]. The potential security vulnerabilities of LMS, particularly for open-source versions such as Moodle, are discussed in more detail in the *Ethics and Cybersecurity in Digital Health Platform* section.

The use of LMS is another area where future developments have been anticipated [31]. Although the original development of LMS such as Moodle had incorporated social constructivist pedagogy [26], there has been a danger that the practical administrative aspects of LMS could predominate. Since the COVID-19 pandemic, this has recently been challenged as an area requiring optimization to move forward [51]. Looking forward, the identities of the 3 main components of a working LMS, namely the learner, the educator, and the LMS system itself, may need to change to establish authentic web-based teaching and learning. In a recent study regarding the LMS Blackboard, only a few of the 53 core study sites analyzed appeared to be at higher levels of identity changes. In essence, most sites were used mainly as digital depository with very limited or infrequent communication from the educators. Such studies have championed the change of LMS from being a predominant interaction between the learner and the LMS to a learning community with educators and learners repositioned as coparticipants [51].

### Telemedicine and Digital Medical Education

Telemedicine allows the transfer of medical services from health professionals where distance may have otherwise been a limiting factor, and it has been used across specialties [52]. In addition, telemedicine has an important role in health education by providing learners an insight into real-world telehealth scenarios and training them in the appropriate medical skills. It is this educational aspect of telemedicine that was considered in this section. The main methods for the delivery of telehealth training in digital medical education range from traditional lectures to simulation-based education [53] and cover a broad range of skills. In the training for telesurgery, for example, medical students can observe procedures and enter associated case-based discussions. In the first decade of the new millennium, despite becoming part of the medical curriculum in France in 2009, teaching telemedicine to medical students elsewhere was still limited [54]. Beyond medical schools, professionals have also seen the benefits of teleconferencing in telemedicine; for example, as a result of distance learning being available, continued professional development opportunities are more accessible to professionals who would normally have been limited by distance, financial burden, or time [33]. Training health professionals often requires more active manipulation of tools with live feedback from remote educators, a form of interactive and immersive teaching method that is generally well tolerated [54]. In addition to ensuring that users are familiar with how to use telemedicine to deliver effective medical care [55], learners should be trained on how best to maintain patient confidentiality and privacy in telemedicine [52,56].

In the late 1990s, training in telemedicine was in its developmental stages [57,58], with its limited use in training at the undergraduate and postgraduate levels [58]. Where training was available, programs were aimed toward practicing medical professionals and were often specific to a form of telemedicine [56,58]. In addition to direct training, telemedicine has been used to provide telementoring, for example, to surgeons in training [59]. In recent years, more medical training curricula have shifted to include training and education on telemedicine [52,58]. By 2019, just before the COVID-19 pandemic, 25% of American medical schools included telemedicine in their curricula [55]. Goals for telemedicine training include improving decision-making and coordination to enable learners in their turn to be able to deliver effective health care [60].

Looking forward, a major challenge for the use of telemedicine in digital medical education is that it cannot fully replicate in-person rapport building, body language cues [52], and clinical skills essential for medical students, despite the efforts of existing nonverbal communication training platforms [61]. Thus, while it has been suggested that the greatest benefits are to be gained when training is commenced in earlier stages of medical education [62], greater integration with traditional teaching may provide the best balance.

Further challenges to anticipate include the expansion of telemedicine for health education more equitably, particularly targeting lower-income countries and rural areas within high-income countries [52,63]. This is particularly important as remote areas in lower-income countries may have the most to gain from telemedicine-oriented digital education [64,65]. Work focusing on sub-Saharan Africa, containing 33 of the 48 lowest-income countries in the world, has provided hope for ways forward to achieve [64,66]. While the first decade of the millennium carried great hope that international African networks, such as the Fundamental of Modern Telemedicine for Africa and the Réseauen Afrique Francophone pour la Telemedicine, might help narrow the gap in telemedicine education across Africa, accessibility is still suboptimal even in 2024 [64]. Ongoing challenges contributing to this include access to the internet, computers, and social media, while only a few medical practitioners were even aware of telemedicine training, as reported in a 2024 study based in Ethiopia [64]. Important factors identified in this study were access to computers and the internet, while a gender discrepancy was identified, where, among the practitioners, there is a greater knowledge of telemedicine among men. Improving existing reimbursement models and fostering more coordinated partnerships between governments, the private sector, and communities in telehealth education have been identified as important steps forward in the coming years [65]. Therefore, rather than additional technological advances, it seems likely that the advances in infrastructure will be one of the key opportunities to further develop the great potential of telemedicine digital medical education in the coming years [65].

### mHealth and Digital Medical Education

The last 25 years have seen a surge in the use of mobile technology worldwide [67-69]. The increased use of mHealth apps [70], particularly within digital medical education, partially reflects the ease of access they can provide. While mHealth apps have often been used for medical practice [71], they have also been widely used in medical education [72,73]. The increased use of mHealth apps for medical education in recent years [74] reflects the call for such expansion in the early 2010s [68]. Examples of mHealth use in digital medical education include web-based textbooks, podcasts, medical calculators, web-based lectures [68,72,73], and internet-based anatomical models used to visualize structures in 3D and medical scans [68]. Simulation apps, such as Touch Surgery [75], have been used to effectively learn, consolidate, and practice surgical maneuvers and clinical skills such as cardiopulmonary resuscitation [76,77]. Furthermore, mHealth apps can facilitate collaborative learning, CME, and clinical decision-making [68,72,73]. In addition to providing education for health care students and professionals, the ease of access to mHealth apps allows them to be used for the health education of patients as well [68]. This educational content can range from teaching patients rehabilitation for chronic obstructive pulmonary disease [78,79] to providing general explanations for their symptoms.

Both within and outside lecture halls and clinics, mobile devices have proven to be a valuable resource for accessing educational content where distance, resources, or financial situations may have previously been a limiting factor [68,73,75,80]. While some apps have paid components [68,76], the low costs of many apps lessen the potential for financial barriers for many learners [68,72]. To maximize the educational benefit and maintain user interest, mHealth apps have implemented strategies, including interactive quizzes, instant feedback, and gamification, which use game-like features, such as point scoring, leaderboards, and challenges to make the learning more engaging [81].

As with many digital health resources, concerns for privacy and confidentiality should be addressed [72]. This can, in part, be mitigated through regular updates of antivirus software; however, the risk of a mobile device being stolen or lost remains. Another challenge may be that the convenience and subsequent reliance on mHealth educational resources could compromise the standard and depth of internal thought that is required in medical practice [72]. Furthermore, physical limitations, such as screen size [68] and obstruction of the device screen during interactive elements [76], can restrict the ways mHealth apps are used for medical education.

Looking ahead, while mHealth apps may be widely available [71,80], a concern among users is regarding the accuracy of results [72]. For example, it was noted in 2012 that >44% of apps giving information on cancer provided data that were not scientifically validated [67]. There is not yet a standardized protocol for establishing the quality of mHealth apps, and the development of such protocols would not only aid design but also allow for more consistent quality control [81]. In the near future, the increasing use of AI, explored more comprehensively subsequently, is likely to simulate complex medical scenarios using mHealth [81].

Further expansion of the reach of mHealth education apps geographically will be an important goal once concerns about equitable access to technology have been addressed. One important aspect regarding this is the need for apps to be available on multiple operating systems [68,81]. Accessibility to mHealth in lower-income nations may superficially appear less problematic than telemedicine, with nearly 4 billion smartphone users recently estimated globally and wide penetration even in many lower-income countries [82]. However, such figures can be deceptive as the full functionality of smartphones requires a good internet connection, which is often problematic in lower-income countries [64]. Similarly, even where rapid uptake of mHealth education apps is accomplished, retention and ongoing participation of users have been more problematic [82]. A recent paper has pointed to the opportunity for improved incorporation of the complexities of learner engagement at the development stage of mHealth apps [82]. This concept has been shown to be effective in a recent study involving the co-design of an mHealth app with the learners, in this case, medical students [83]. Building on such personalized models for mHealth learners in the future could more closely align with the use of similar apps designed for patients [68] and may lead to greater engagement and agency among learners.

### BDA in Digital Medical Education

Through ever-increasing interactions with digital technologies, users generate large amounts of data. BDA describes the process of collecting and interpreting these large datasets from multiple sources to gain meaningful information [84]. The history of big data may be outlined in terms of the volume of typical datasets over time. The late 1990s, at the beginning of our chosen review period, have been considered the milestone when the terabyte (a billion bytes of data) started giving way to petabytes (1000 terabytes), while the second decade of the new millennium has been viewed as the transition from petabytes to the exabyte (1000 petabytes) [85]. BDA use is widespread across industries and has been associated with reduced costs, improved productivity, and an impact on decision-making [86]. In the last decade, developments have been made in the use of BDA in health care, supporting improvements in patient monitoring, data-driven diagnosis, management of hospital resources to improve service quality, and evidence-based decision-making at both a public health policy level and a local level [87]. However, applications of BDA in digital medical education have only begun to accelerate in more recent years [88]. BDA holds great potential to enhance learning, although a recent systematic review showed that analytics were most frequently used to simply capture the number of connections made by students to the learning material, while areas such as feedback and at-risk intervention were yet to be fully used [89].

A key quality of BDA lies in its ability to support decisions. Changes to curricula, course structures, and admission policy can be visualized by their impacts on performance data [90], allowing program directors to swiftly notice patterns and respond to them. In addition, accumulating performance data could help generate benchmarks for programs as an additional metric for ensuring quality standards. On an individual level, BDA can help to identify or predict students and trainees who are struggling or at risk of underachieving, allowing extra intervention to be provided for them [91]. However, there has been some debate regarding the psychological consequences of using predictive analytics in this way and whether it may potentially exacerbate a student’s struggles by prematurely labeling them as falling below a certain metric [92].

Furthermore, digital medical education platforms and institutions may use BDA as a form of feedback by using data patterns on engagement instead of relying on manual questionnaires, helping to save time and potentially producing more valuable insights into where improvements need to be made in health education [93]. For example, data on whether medical students engage with digital content (eg, clicking hyperlinks or magnifying images on course web pages) have demonstrated value in predicting which students would perform better at examinations [94]. With focused use, BDA can facilitate and optimize learning methods, helping learners to understand specific medical content and aiding in the personalization of the education process. Performance data on clinical skills, such as catheter insertion, can create a baseline to judge when a student is ready to perform such skills on a real patient while also providing the student with reassurance and confidence when they are ready [90]. Data collected about learners should be fed back to them, closing the loop, and allowing them to have a greater understanding of how data are being used and how they will benefit them [95]. One way of doing this is by creating personalized dashboards for students, which systematically deliver and help visualize performance data [96].

Looking to the near future, an ambitious endeavor of BDA may be possible from the combination of data on clinical information in hospitals with data from medical education, to gain an insight into how different medical curricula and educational models impact patient outcomes [90]. However, it may be difficult to interpret these data-generated patterns, as many other factors can affect clinical outcomes, including team decisions, local practices, and technological disparities, making it hard to attribute results to individual educational programs [97]. Other challenges associated with BDA still require consideration. Emerging patterns have been shown to be open to misinterpretation, as data may simply be correlated and not causally linked [90]. In addition, as with other large datasets, there is the potential trap of finding examples of significant patterns that may not necessarily be meaningful [98]. Traces of a learner’s digital data, reflecting their behaviors on the web, may potentially be gathered as part of big data when using digital health technologies in education [99]. It is still unclear what the exact limits on the use of such data would be in the future; however, it is an important area to be cognizant of, given the increased use of such technologies in digital medical education [100].

### The Metaverse, AR, and VR in Digital Medical Education

The metaverse is a 3D web-based world in which users can interact both with other users and with digital objects, often, but not exclusively, experienced through AR and VR [28]. While the metaverse is not in itself a new concept, the more recent rapid development of associated tools, such as blockchain and nonfungible tokens as well as the close association with the evolving Web 3.0 in the last decade [101], have created considerable opportunities across digital environments, particularly in digital medical education. The decentralized concept of Web 3.0 provides the potential for greater dissemination of education while also posing challenges for education providers in terms of possible loss of some control over content and ensuring that legal procedures, from data protection to copyright and accessibility laws, are strictly followed. At the same time, individual educators and educational companies now have increasing opportunities to interact with learners directly using the blockchain features of Web 3.0 to monetize the services they provide, potentially bypassing traditional educational institutions [28].

Earlier integrations of the metaverse in medical education involved the use of web-based classrooms, where avatars represent and perform actions on behalf of the user. These 3D worlds have been reproduced on a computer monitor without the need for headsets or lenses. A platform exploring these internet-based worlds for digital medical education includes Second Life, developed by Linden Lab in 2003, which provides educational materials on various medical topics and offers consumers access to health information [102]. Another promising platform for integrating metaverse platforms into medical education is the open-source OpenSimulator project [103]. This platform has provided teaching in multiple medical specialties, with base models that have been repurposed and customized to create web-based medical center scenarios.

As the metaverse is built around extended reality technology aimed at making an immersive experience for users, the more focused terms AR and VR are important to consider [28]. AR involves a digital overlay on top of the real world, whereas VR involves full immersion into the digital sphere [104]. Both AR and VR can be realized via head-mounted devices [105] or digital glasses [106], among other technologies, and represent just a few of the many ways in which the metaverse is experienced. An important distinction between the metaverse and some of the experiences offered by AR and VR lies in the nature of the interactions with other humans. While the metaverse is inherently social, providing a shared digital environment for multiple users, AR and VR can also enable an individual to interact with immersive environments independently without the need for social contact.

AR has shown significant value in training techniques across numerous medical specialties and is seen frequently in surgery [107]. For example, as a urological surgery simulation tool, Google Glass demonstrated positive outcomes in medical students and urological surgeons across various stages of training [106]. This training provided a sense of reality in the training environment, which was particularly favored by the younger participants. AR has also shown benefits for students studying anatomy, with AR textbooks assisting students in learning more effectively compared to standard textbooks [107]. Furthermore, students taught with AR were found to have better performance in long-term spatial anatomy knowledge compared to those taught traditionally [107].

In terms of the efficiency of VR, a randomized controlled trial carried out with Japanese nursing students noted no significant differences between individuals using VR and those taught with traditional methods in terms of skills, knowledge, or confidence [108]. The only statistically significant finding was a higher satisfaction rate in students who were taught traditionally, likely due to the sickness associated with VR adjustment. The study was limited to a small sample size and only tested third-year students, making it difficult to determine its wider applicability. Similar results were seen in a study on undergraduate paramedic students, with no significant differences between the results of those who had lecture-based teaching and those who had VR-based teaching [109]. However, a systematic review evaluating immersive VR use in nursing education [110] highlighted that there is generally a positive response to VR-assisted learning among nursing students. This included reduced anxiety in those performing skills in real life with prior VR practice due to 360-degree simulations that resembled real life more closely than simulation situations with manikins or lectures. A systematic review conducted by Foronda et al [111] found similar results, with the efficiency of VR typically not being statistically different from traditional teaching methods. However, it was also noted that most trials using VR only used it as an intervention once, and there was a lack of data showing how technology faired to traditional teachings across multiple sessions. VR simulators have shown significance compared to controls in providing greater self-confidence for surgical residents in dentistry [112]. Furthermore, it was identified that residents who used VR navigation software had higher accuracy in marking their first implantation sites during the surgical stage of dental implantation [112]. A recent meta-analysis performed by Kyaw et al [113] showed that teaching health professionals with VR interventions that are mostly interactive resulted in a considerable improvement in skillset and, to a lesser extent, improvement in knowledge compared to traditional methods. Both AR and VR are promising as active learning methods for teaching anatomy to undergraduate students. Combining these methods into extended reality teaching was perceived by health care students as more useful than traditional methods [114].

There are some device-related downsides to both AR and VR, as they are primarily dependent on the associated headsets or digital glasses, and device development in the coming years should consider these issues. For example, users who already wear spectacles report discomfort when wearing such devices on top of them [106]. Device use may also be associated with nausea, motion sickness (sometimes termed “cybersickness”) [104], technical difficulties, and stress, and it is unclear whether such symptoms resolve as a participant becomes accustomed to the experience over time or whether they are long-lasting symptoms [104,105]. There is a need to develop clear guidance for potential users of AR and VR to determine whether any preexisting health conditions might need additional support or to take precautions before using these devices [104]. Notably, currently, there is a lack of universal standardization or established guidelines encompassing VR, the metaverse, and AR.

AR and VR devices currently require regular charging, which can be inconvenient when needed for long periods and may benefit from future improvements in battery technology. Furthermore, there are concerns about accessibility to such education in low- and middle-income countries, despite having populations that might particularly benefit from AR- and VR-based education due to the unequal distribution of health professionals. Inequalities and limited accessibility have been noted in many countries, including higher-income ones, with internal disparities between urban and rural areas [105]. As mentioned in the *Telemedicine and Digital Medical Education* section, lower-income countries and isolated rural areas, even in higher-income countries, may stand to benefit the most from the metaverse if it becomes accessible in more remote areas in the near future. As technology and equipment become more readily available, the overall cost of AR and VR should decrease, improving accessibility in low- and middle-income countries.

There are some scenarios where students’ confidence in AR or VR education techniques may be misplaced, as a systematic review by Baniasadi et al [104] suggested that a challenge of remote VR education may be a lack of direct supervision and evaluation of student performance. In addition, there may be limited technical support for those who may find it difficult to manage the complexity of VR independently. Increasing student engagement with AR or VR teaching by gamifying digital learning materials may also increase social connectivity, helping combat the risk of social isolation in remote learning, and seem likely to be important areas for expansion in the future [115].

### Impact of the COVID-19 Pandemic on Digital Medical Education

The COVID-19 pandemic, which began in early 2020, impacted nearly 1.6 billion learners, that is, 94% of all students worldwide at the time, with enforced social distancing necessitating rapid and significant changes in education, particularly with a pivot from face-to-face to remote digital delivery [116]. For many educators, who might previously only have been accustomed to teaching face-to-face, the rapid change required to convert to distance learning with digital delivery created tremendous challenges [117]. The rapid upskilling of these individuals in fundamental digital skills, such as the use of videoconferencing, was vital across many courses globally, including in health education. For most educators, the immediate practicalities of rapidly upskilling educators and changing delivery to digital formats had to take precedence over exact pedagogies [116]. Preexisting providers of digital medical education were in a position to help their peers adapt. There were significant challenges faced by students as well [118]. For those who had originally applied for completely face-to-face courses, the experience of distance learning may have differed significantly from what was anticipated. We should not underestimate the very significant added stresses of the time [119]. For example, in many countries, lockdowns added restrictions on movement and concerns about risk to personal health or the health of loved ones. This multitude of factors makes it difficult, even in retrospect, to fully analyze the effectiveness of digital medical health education during the pandemic. However, what does seem certain is that the COVID-19 pandemic was associated with a much wider subsequent dissemination of digital health teaching [120].

Much of the early literature published during the time of the COVID-19 pandemic focused on the adaptations used by medical health educators to pivot to the digital environment. Many of these specifically looked at the practical steps that were required, including training on acceptable behaviors expected during these digital meetings, especially using videoconferencing [120]. There appear to be more studies conducted in the United States that report the direct use of web-based discussions with a patient as part of the digital education delivery to students compared to those in the United Kingdom and Europe [120]. Prolonged use of videoconferencing was associated with learner fatigue, in some cases, postulated to stem from critical self-analysis of the learner’s face appearing on the screen during teaching [121].

The enforced change of educational delivery to digital platforms away from classrooms may have risked some social isolation, especially in university student populations where much socialization occurs during in-person classes [118]. Various techniques have been used during videoconferencing to help improve focus and engagement between students and educators and among students themselves. Regular chunking of information and the use of breakout rooms may have benefited some in this regard. With apparently less forthcoming students, sometimes referred to as “lurkers” [120], these techniques may have been effective. The interactive behavior of web-based learners has sometimes been sharply divided into “the workers, the lurkers, and the shirkers” [122]. However, the role of more passively “lurking” in the background during videoconferencing calls may, in fact, be used by most attendees early on, as they adjust to the meeting and before they can participate more fully [123].

Despite the challenges of the time, student surveys during the pandemic revealed some positive feedback regarding their enforced web-based medical education [124]. Since the last of the lockdowns in most countries was lifted, beginning in March 2021 in England [125], the evidence overall indicates that there has been increased use of digital education methods since prepandemic times [120]. For example, in the United States, the total number of students enrolled in degree-granting postsecondary institutions who had ≥1 of their courses delivered on the web was >14 million (75% of the total) at the height of the COVID-19 pandemic in autumn 2020, but this number was still >11 million (60% of the total) in autumn 2021, well after nationwide lockdowns had been lifted [126]. This was still substantially above the prepandemic 2019 level when 42% of postbaccalaureate (postgraduate) students were enrolled in ≥1 distance education course [127].

Many learning institutes had not only upskilled educators but also invested in the technology to permit digital learning [128]. In addition, the sudden enforced changes in education during the COVID-19 pandemic may have made changes in previously accepted higher-education structures easier to gain ground. For example, we are noticing a rapid rise since the COVID-19 pandemic in alternative courses to formal higher-education degrees, such as microcredentials and digital badges, with which digital medical education could be used to offer greater flexibility to learners than with traditional degree courses, even compared to those with a modular structure [129].

### AI in Digital Medical Education

The term “artificial intelligence” (later abbreviated as AI) was first coined in 1955 and loosely described machines that could use language to solve problems, create abstract concepts, and improve themselves in doing so [130]. However, it is in the last decade that dramatic developments in this field have led to both great potential and great challenges for digital medical education. AI has been used in areas as diverse as medical practice management, patient monitoring, diagnostics, and device integration and within medical education in a range of fields, including radiology, dermatology, and surgery [131,132]. The use of AI tools in digital health has been particularly impactful, with AI tools demonstrating the capacity to outperform even highly experienced professionals, highlighting its potential to reshape health care education by optimizing training and providing decision support tools [133]. Despite this, the use of AI in digital medical education has, to date, lagged somewhat behind its use in medical practice.

Large language models, such as ChatGPT, have become very widely used in the last 2 years across many disciplines. Despite several potential challenges, they can quickly offer a very brief summary of many long-established facts in medicine [134]. Consequently, they can be used partially when preparing medical education, including the generation of patient cases and practice questions. Because of plausible errors and dataset time limitations, the current use of such large language model studies may be less effective for students than experienced educators who could more easily spot errors. Examples of how medical educators might use tools, such as ChatGPT, have included internet-based patient simulation, quizzes for medical students, summarizing research articles, and generating brief curricula for digital health learners [134]. However, the rapid production of relevant material by large language models gives rise to concerns that some students might use them to help answer examination questions. In 2023, it was shown that ChatGPT could perform as well as a typical third-year medical student on Step 1 and Step 2 in the US Medical Licensing Examination, and further performance improvements are likely [135]. Nevertheless, students might benefit from interacting with ChatGPT in a dialogue to explore concepts when learning independently or with peers [135]. It is currently not possible to detect with certainty whether a student who submitted their work had used AI to answer examination questions, and this is especially a concern with noninvigilated examinations. While tools such as the generative textual likelihood ratio can help humans detect work produced using large language models, the high stakes of plagiarism for students can make even 99% accuracy seem insufficient to confidently declare that AI models were used [136]. Hence, teaching institutions have to reconsider the assessment environment for students with invigilated face-to-face or distance learning examinations in real time. Furthermore, alternative approaches, such as higher marks for critiquing, use of modern references, or interpretation of medical images by students, may currently help, but even these may become less challenging for large language models in the near future [137]. A similar concern in digital medical education extends beyond students. A recent study demonstrated that it was possible to use ChatGPT (GPT-3) to deliberately produce a fraudulent paper nearly 2000 words long with 17 references in just 1 hour. While there were some errors, such as false references and so-called “hallucinations,” [134] which an expert might detect, this detection rate may decrease with time as large language models continue to improve.

AI text-to-image generation of high-quality images depicting physical appearances has proved possible with the addition of diffusion models over the last decade, allowing the potential production of illustrations even of rare medical conditions [138]. For example, images of arthritis, a potential thyroid mass, and hypothyroidism have been produced as entirely novel images not based on traditional photographs of patients [139,140]. The images of the face, in particular, may offer great promise as an alternative to traditional patient photographs, where the very important issues of consent and confidentiality may limit the availability of images. Such AI text-to-image generation can also rapidly produce novel pictures to illustrate case studies for teaching. Moreover, many tools are now widely available to produce new, short videos from AI text-to-video prompts. However, most tools to date mainly use speaking avatars (with some degree of apparent lip-synching) on a background of very short traditional stock video clips, which are typically linked together via AI to create a finished video. These tools certainly have the potential to enliven a short text teaching session. There is also rapidly evolving work on creating genuinely new moving images without the use of stock videos. OpenAI, in early 2023, demonstrated some high-quality short videos using Sora, which may be more widely available later in the year and potentially then be used for digital medical education [141].

Looking forward, pressing challenges to address regarding AI include clarifying the copyright of AI-generated text and images, including the limits of such images and the lack of clarity about the original dataset of images used to train the generative adversarial networks through which AI image-generating tools are developed. Other potential issues, such as accuracy, and potential perpetuation of stereotypes (including skin color and perceived gender) have been raised [142]. Similar concerns have been raised for text-to-video generation, with the possibility that inaccurately portrayed medical images or deliberately misleading videos (including “deep fake” images) may be produced [141]. The establishment of a global quality control body of AI-generated products could substantially help to manage challenges such as these. Watermarking of work produced using large language models and text-to-image generation began in 2024, although this may not be impregnable, and further development in the coming years is expected [136]. Despite these considerable cautions, the field of AI text-to-image and text-to-video generation seems to have great potential for the near future of digital medical education while recognizing the important challenges that have been mentioned in this field. This marks AI as one of the biggest potential areas of growth in digital medical education looking to the immediate future.

### Ethics and Cybersecurity in Digital Medical Education

The ethical and legal considerations of digital medical education have not been explored in extensive depth in the currently available published literature; however, some extrapolation is possible from work looking primarily at digital health and is highly relevant given the many advances in digital education in the last 25 years. A recent review found that disparities in digital access, especially among users with different ethnicities or income statuses, contributed significantly to eHealth use and exacerbated social health inequalities [143]. Likewise, digital medical education may be a disproportionately less available option for racial or ethnic minority groups, older adults, and those with limited health literacy [143]. This raises concerns regarding the fairness of accessibility of educational resources across all demographics. The open access model, used successfully in many medical journals, might provide a model to follow while trying to develop greater equality digital medical education, potentially building on some of the challenges that still exist in some areas of open access [144].

Health organizations do not always have the level of cybersecurity needed to prevent unwarranted access to personal patient information [145]; therefore, educational content created from patient information or sensitive data may not be appropriate to use without adequate safeguards. This should be borne in mind when obtaining consent from patients for the use of such material (eg, medical photography) in education. Safeguards should be put in place to restrict the inappropriate distribution of such content to unintended personnel via means such as social media [146]. This is particularly the case in digital education, where images and videos might be stored for long-term use, including downloading, manipulating, and uploading [147]. The use of encryption software may aid in securing data with sensitive information, with multiple layers of encryption providing stronger protection [148]. This may help overcome some ethical concerns regarding data storage, as not only text but also some photos and videos can be anonymized. General Data Protection Regulation compliance is important in digital medical education, with aspects such as obtaining informed consent data minimization, and anonymization, all being factored in [149]. Securing password protection of accounts and software or creating personalized links for individuals may help ensure that data are only accessed by people with given log-ins, helping to regulate those who can access educational software that includes patient images or data. In addition to ensuring that data protection protocols are followed, it is also fundamental that the significance and impact of data protection is taught to health care students who are increasingly accessing health and education digitally. However, it is currently unclear how widely digital health competencies have been achieved among health workers in dealing with aspects such as electronic health records [150,151].

The immediate future holds significant cybersecurity concerns. Potential security breaches can reach any area of digital health care courses, even its fundamental learning platforms. For example, concerns about potential vulnerabilities of LMSs, especially open-source versions such as Moodle, have been highlighted. As LMSs are used to form the main foundation of many digital medical education courses, this is a key area that will need ongoing vigilance [26,31]. As Moodle was designed to store user data in caches, these could theoretically be targeted by an attacker to launch an attack with the subsequent session [26]. Even with limited chances to access the system, brute force cyberattacks have also been highlighted as a potential vulnerability [26].

Digital medical education platforms may have some potential vulnerabilities, which have been categorized as falling into 4 main groupings [26]. Authentication challenges may occur if an attacker uses the “forgot your password” option. If session tokens are sent in response without proper encryption, security may be breached. Availability issues may involve denial of service instigated by either flooding or logic attacks. Confidentiality attacks attempt to access existing confidential data on the learning system, while integrity attacks attempt to modify or delete stored data [26]. Suggested improvements, including the use of Completely Automated Public Turing test to tell Computers and Humans Apart when logging in to help counter brute force attacks and the use of a secure sockets layer to help prevent hijacking of sessions, have not yet been fully implemented, and increasing the use of such technologies in the immediate future would assist with digital course security [26]. While such technical improvements would further enhance digital security, it seems likely that human error is important in many cybersecurity breaches as with digital health care [152]. Therefore, further developments of tailored educator training to avoid breaches are likely to be an effective approach in countering the increasing risk to cybersecurity [152].

One possible risk of the increasing use of digital medical education is that it might contribute to digital addiction, potentially affecting 25% of the general population, especially in instances of the metaverse, AR, and VR being integrated into health education through game-like apps [153]. There are concerns that technology overuse due to the introduction of digital medical education might exacerbate social isolation and reduce opportunities for health care students to benefit from the positive role modeling of professional behaviors, such as empathy and communication skills [118]. Although student response systems, such as Socrative, use games to improve learner engagement, Kahoot! was the first to be designed based on a theory of intrinsic motivation [154]. Kahoot! was only realized as a platform in 2013; however, by 2019, >2.5 billion individuals had played it, underlying the great importance, both of potential benefits and ongoing vigilance to avoid potential digital addiction with student response systems [154].

## Discussion

### Innovations and Future Directions

Digital medical education has evolved dramatically in the past 25 years. While we have examined key components of this journey, some overarching themes emerge. Overall, a paradigm shift has occurred during this time, seeing a change from didactic, expository digital medical education to more active learning models. Technologies incorporated during this time include LMS, medical simulation and role-playing with VR and AR, and the metaverse. The resulting advancements in ways that medical education can be created, managed, and experienced have offered pointers for further possible developments in digital medical education. The increasing use of the internet and mobile apps are just a few examples of the many technological advancements that are reshaping how health care professionals are educated, increasing learning efficiency and offering uniquely personalized learning experiences. Digital medical education continues to expand before us, propelled by the rapid recent advances in these digital tools and underlying components, such as AI.

Developments in digital medical education have seen significant growth in recent years, driven by the emergence of generative AI and advancements in AR and VR, which have enabled immersive, simulation-based learning. Looking to the future, we can anticipate further integration of AI-associated learning systems; the use of wearable technology to provide more immersive experiences and real-time feedback; and the use of blockchain, for example, to store, encrypt, and verify certifications. Furthermore, future synthesis of AI may include improved personalized analytics for students, shaping an era where students have a more transparent understanding of their progress, competency in learning a skill, and their personal learning styles.

### Challenges and Opportunities

However, several key challenges require ongoing attention. These include the need for improved accessibility to relevant technologies and, in some areas, improved internet connectivity. Ethical challenges also exist on several fronts, especially surrounding consent, confidentiality, and ownership of the web-based material used in digital medical education. While a boon for many, this relentless progress in digital medical education may leave some learners, and even some educators, with a feeling of being unable to keep pace with the evolving digital landscape, causing fear and stress [119,120]. To address this, institutions should prioritize accessible training programs, mentorship opportunities, and user-friendly interfaces to build confidence and competence.

Despite concerns about the rapid advances in technologies underlying digital medical education today, these changes should be embraced rather than feared, giving us further opportunities to support and empower our learners. However, at its heart, digital medical education will always be more than the technology and rather should enhance ways that we, as educators, can reach, support, motivate, and empower our learners.

## Abbreviations

AI: artificial intelligence
AR: augmented reality
ARPANET: Advanced Research Projects Agency Network
BDA: big data analytics
CME: continuing medical education
GP: general practitioner
LMS: learning management system
mHealth: mobile health
MOOC: massive open online course
PLATO: Programmed Logic for Automated Teaching Operations
VR: virtual reality
